# Potential of Titanium Pins Coated with Fibroblast Growth Factor-2–Calcium Phosphate Composite Layers to Reduce the Risk of Impaired Bone–Pin Interface Strength in the External Fixation of Distal Radius Fractures

**DOI:** 10.3390/jcm13113040

**Published:** 2024-05-22

**Authors:** Hirotaka Mutsuzaki, Yohei Yanagisawa, Hiroshi Noguchi, Atsuo Ito, Masashi Yamazaki

**Affiliations:** 1 Center for Medical Science, Ibaraki Prefectural University of Health Sciences, Ami 300-0394, Japan; 2 Department of Orthopedic Surgery, Ibaraki Prefectural University of Health Sciences Hospital, Ami 300-0331, Japan; 3 Department of Emergency Medicine, University of Tsukuba, 1-1-1 Tennodai, Tsukuba 305-8575, Japan; yanagisawa@tsukuba-seikei.jp; 4 Department of Orthopaedic Surgery, University of Tsukuba, 1-1-1 Tennodai, Tsukuba 305-8575, Japan; noguhiro0164@tsukuba-seikei.jp (H.N.); masashiy@md.tsukuba.ac.jp (M.Y.); 5 Health and Medical Research Institute, National Institute of Advanced Industrial Science and Technology (AIST), AIST Tsukuba Central 6, 1-1-1 Higashi, Tsukuba 305-8566, Japan; atsuo-ito@aist.go.jp

**Keywords:** fibroblast growth factor-2 (FGF-2), calcium phosphate, coating, external fixation, distal radius fractures, impaired bone fixation, Weibull plot analysis

## Abstract

**Background**: The risk of impaired bone–pin interface strength in titanium (Ti) pins coated with fibroblast growth factor (FGF)–calcium phosphate (CP) composite layers is yet to be evaluated in a clinical study. This retrospective study used Weibull plot analysis to evaluate bone–pin interface strength in Ti pins coated with FGF-CP layers for external distal radius fracture fixation. **Methods**: The distal radial fractures were treated with external fixation. The FGF-CP group comprised five patients (all women, aged 70.4 ± 5.9 (range: 62–77) years), and the uncoated pin group comprised ten patients (eight women and two men, aged 64.4 ± 11.7 (range: 43–83) years). The pins were removed after six weeks. The insertion and extraction peak torques were measured. The extraction peak torque was evaluated using Weibull plot analysis. **Results**: We compared the extraction torque of the two groups at or below 506 Nmm for a fair comparison using Weibull plot analysis. The Weibull plots were linear for both the FGF-CP and uncoated pin groups. The slope of the regression line was significantly higher in the FGF-CP group (1.7343) than in the uncoated pin group (1.5670) (*p* = 0.011). The intercept of the regression line was significantly lower in the FGF-CP group (−9.847) than in the uncoated pin group (−8.708) (*p* = 0.002). Thus, the two regression lines significantly differed. **Conclusions**: Ti pins coated with FGF-CP layers exhibit the potential to reduce the risk of impaired bone–pin interface strength in the external fixation of distal radius fractures.

## 1. Introduction

Screw loosening is a severe clinical problem in orthopedic surgery that leads to unfavorable clinical results, including incomplete healing of bone fractures and delayed union [1,2,3]. Plasma-sprayed apatite coating is known to increase the extraction torque of external fixation pins compared with that of uncoated pins [4,5]. Calcium titanate screws have an increased fixation index, which is the quotient of maximum extraction torque over maximum insertion torque for external fixation compared with uncoated pins [6]. Bisphosphonate coatings for external fixation in metaphyseal fixation strength are similar to hydroxyapatite coatings [7]. In these studies, average values of bone–pin interface strength were compared between treated pins and untreated pins. However, an average value does not necessarily reflect an incidence probability of an outlier, such as a very low value of bone–pin interface strength, since an outlier rather relates to dispersion.

We developed titanium (Ti) screws coated with human recombinant fibroblast growth factor (FGF)-2–calcium phosphate (CP) composite layers by immersing them in an infusion fluid-based supersaturated CP solution containing FGF-2 at 37 °C for 48 h [8]. The risk of impaired bone apposition to the screw was analyzed using Weibull plot analysis, a method commonly employed to analyze the lifetime, failure probability or risk, and reliability of industrial products [9]. As FGF-2 is a human recombinant protein, pedicle screws coated with an FGF-CP layer were implanted in cynomolgus monkey spines to simulate potential clinical use [8]. The pedicle screws coated with FGF-CP layers exhibited a significantly lower risk of impaired bone formation, as analyzed using Weibull plots [8]. In a clinical trial, Ti pins coated with FGF-CP layers in external fixation of distal radius fractures demonstrated safety and a tendency towards a reduced pin tract infection rate [10]. However, it is unclear whether the Ti pins coated with FGF-CP layers reduce the risk of impaired bone–pin interface strength in clinical studies. Using Weibull plot analysis, the purpose of this study was to retrospectively analyze the clinical trial data to evaluate the risk of impaired bone–pin interface strength in pins coated with FGF-CP layers. We hypothesized that Ti pins coated with FGF-CP layers would reduce the risk of impaired bone–pin interface strength that leads to pin loosening.

## 2. Materials and Methods

### 2.1. Participants

Fifteen patients who had fractures of the distal radius with unstable and displaced fragments were enrolled [10]. Fractures were treated using external fixation [10]. In the FGF-CP-coated pin group, five consecutive patients (all women, aged 70.4 ± 5.9 (range: 62–77) years) were enrolled between February 2013 and January 2015 [10]. In the uncoated pin group, ten consecutive patients (eight women and two men, aged 64.4 ± 11.7 (range: 43–83) years) were enrolled between January 2015 and August 2017 [10]. The exclusion criteria were as follows: patients with skin disease, a severe systemic disease (heart, lung, liver, or kidney disease, etc.), a malignant tumor within 5 years before the fracture, pregnant, and who were determined by the doctor as inappropriate [10].

### 2.2. Study Design

This study was a retrospective study that analyzed biomechanical data from the previous open-label controlled feasibility studies [10].

### 2.3. FGF-CP Coating Technique

**Figure 1:**
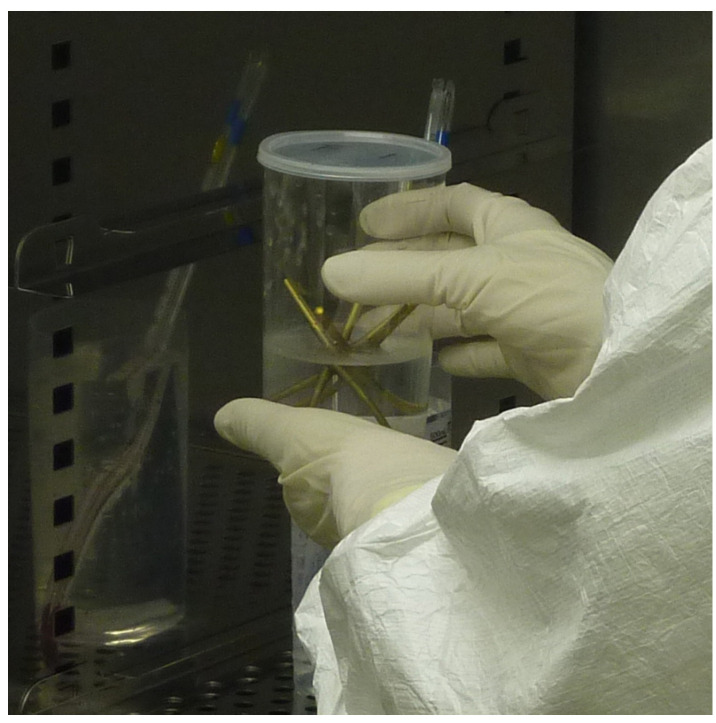
FGF-CP-coated pins.

Ti pins were immersed in a supersaturated calcium phosphate solution containing FGF-2 (4.0 μg/mL) at 37 °C for 48 h under air cleanliness condition class 5 using a clean bench in a clean room (class 6) [10] (Figure 1). The Ca/P molar ratio was 1.67 [10]. The layers retained their FGF-2 mitogenic activity, examined by fibroblastic NIH3T3 cell proliferation [10]. All the supersaturated CP solutions were aseptic, revealed by the bacteriologic and endotoxin tests [10].

### 2.4. Operation and Measurement Procedures

**Figure 2:**
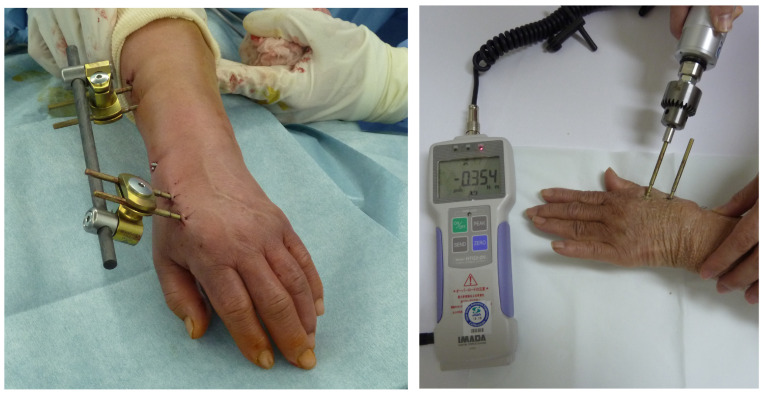
External fixation for fractures of the distal radius. Immediately after surgery (**left**) and extraction peak torque measurement (**right**).

All operations were performed with external fixation using an external distal radius fixator (bridging and unilateral types: DePuy Synthes, Zuchwil, Switzerland), following the original operation manual to standardize pin insertion techniques [10]. Two pins were inserted into the radial shaft through a 10 mm incision, while the other two pins were inserted into the second metacarpal [10] (Figure 2). After six weeks, the pins were removed [10] (Figure 2). The insertion and extraction peak torques were measured using a digital torque wrench (HTG2-5G; IMADA, Toyohashi, Aichi, Japan) [10].

### 2.5. Weibull Plot Analysis

The extraction peak torque was analyzed using Weibull plot analysis according to the following Weibull equation:

lnln [1/(1 − *S*)] = *m* ln *σ* − *m* ln *ξ*,

where ln, *S*, *m*, *σ*, and *ξ* indicate the natural log, failure probability, Weibull parameter, extraction peak torque, and scale parameter, respectively. Thus, the plot of “ln *σ*” against “lnln [1/(1 − *S*)]” gives a straight line with a slope of “*m*”. In this study, *S* is the probability of obtaining an extraction peak torque at or less than *σ*. The measured *σ* values were arranged in ascending order, such as *σ*_1_, *σ*_2_, *σ_j_*, and *σ_N_*, where *j* is the order of an individual *σ* value and *N* is the total number of measured *σ* values. *S* was derived from the median rank method using *S_j_* = *(j* − 0.3)/(*N* + 0.4).

### 2.6. Statistical Analysis

Student’s *t*-test was used to evaluate statistically significant differences. The level of significance was set at *p* < 0.05.

## 3. Results

**Figure 3:**
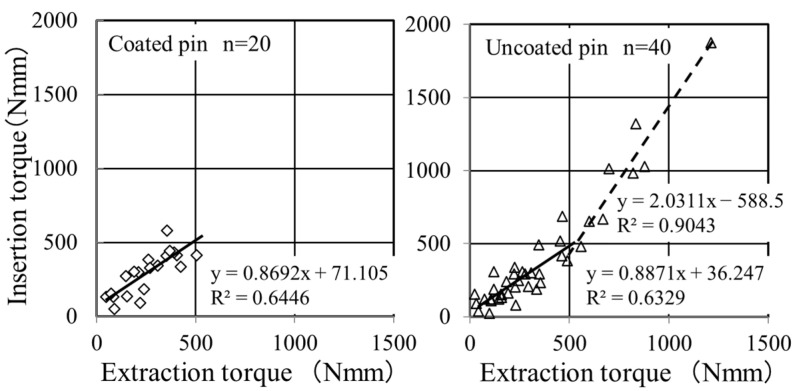
Relationship between the insertion and extraction torques. FGF-CP (**left**) and uncoated (**right**) pin groups.

Figure 3 shows the relationship between insertion and extraction torques. The FGF-CP group had the highest value of 505 Nmm in extraction torque. The uncoated pin group showed a bimodal correlation between insertion and extraction torques, with a boundary at approximately 500 Nmm in extraction torque. Above the boundary, eight of the twelve points belonged to the three youngest patients (patients C1, C3, and C6 in [10]). The slope and intercept of the regression line above the boundary significantly differed from those below the boundary (slope: *p* = 9.7 × 10^−6^, intercept: *p* = 1.3 × 10^−4^) and those for the coated pin group (slope: *p* = 1.1 × 10^−4^, intercept: *p* = 2.6 × 10^−4^). Conversely, no significant difference was noted in the slope and intercept between the regression line below the boundary and those for the FGF-CP group (slope: *p* = 0.46, intercept: *p* = 0.26). Thus, we compared the extraction torques of the two groups at or below 506 Nmm for a fair comparison using Weibull plot analysis.

**Figure 4:**
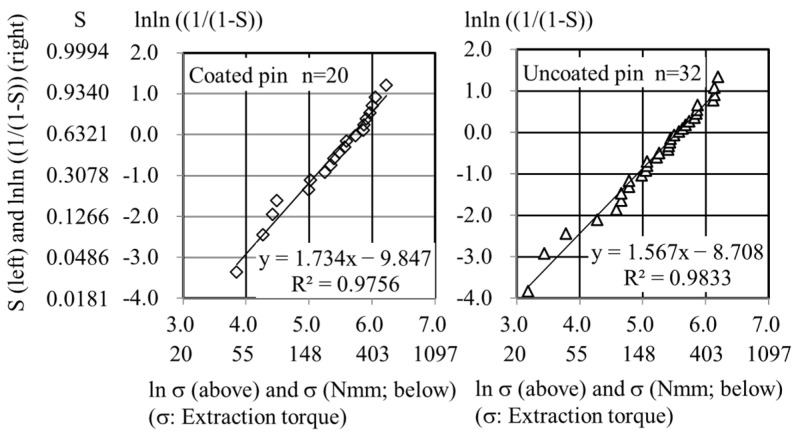
The Weibull plot of extraction torque for the FGF-CP (**left**) and uncoated pin (**right**) groups.

The Weibull plots were linear for both the FGF-CP and uncoated pin groups (Figure 4). The slope of the regression line was significantly higher in the FGF-CP group (1.7343) than in the uncoated pin group (1.5670) (*p* = 0.011). The intercept of the regression line was significantly lower in the FGF-CP group (−9.847) than in the uncoated pin group (−8.708) (*p* = 0.002). Thus, the two regression lines exhibited a significant difference.

The regression lines enabled us to assess the risk of impaired bone–pin interface strength by calculating the probability of obtaining specific low values of the extraction torque, which were arbitrarily selected. For instance, if the impaired bone–pin interface strength was defined as an extraction torque (*σ*) at or less than 20 Nmm, then “ln *σ*” gives a value of 3.0 (Figure 4). Since “ln *σ*” is “x” of the linear regression function, “ln *σ* = 3.0” gives y values of −4.651 and −4.013 for FGF-CP and uncoated pin groups, respectively. Since “y” is “lnln ((1/(1 − *S*))”, one can obtain the failure probabilities, *S*, of 0.9% and 1.8% in the FGF-CP and uncoated pin groups, respectively. Similarly, if the impaired bone–pin interface strength was defined as an extraction torque of ≤100 Nmm, the probabilities were 14% and 20% in the FGF-CP and uncoated pin groups, respectively. Thus, the risk of impaired bone–pin interface strength was lower in the FGF-CP group than in the uncoated pin group.

Similarly, the Weibull plots for all the 40 extraction torques in the uncoated pin group had a regression line with a slope that was significantly lower (1.340; *p* = 1.2 × 10^−8^) than that in the FGF-CP group (Figure S1). This again showed that the risk of impaired bone–pin interface strength was lower in the FGF-CP group than in the uncoated pin group.

## 4. Discussion

The risk of pin loosening is lower in the FGF-CP group than in the uncoated pin group, as shown by Weibull plot analysis. Stability of the bone–pin interface is achieved in the FGF-CP group. The Weibull plots are analyzed with the linear regression of plots (Figure 4). The greater the slope of the regression line is, the lower the probability of failure is, meaning that a more consistent treatment outcome is potentially obtained. When the slope is the same, a lower position of the regression line corresponds to a lower probability of failure. In the Weibull plot of extraction torque regression for the FGF-CP group, the line exhibits a greater slope and runs at a lower level compared to that of the uncoated pin group. This indicates that FGF-CP demonstrates a more consistent treatment outcome and a tendency to reduce the probability of impaired bone–pin interface strength in comparison to the uncoated pin group.

The mode of action on bone–pin interface strength is different between hydroxyapatite-coated and FGF-CP-coated pins. The average bone–pin interface strength for hydroxyapatite-coated pins is higher than that of uncoated pins [4,5,11,12,13,14]. In general, the higher bone–pin interface strength in average led to low incidence rates of impaired bone–pin interface strength [15,16]. In some reports, the incidence rates of impaired bone–pin interface strength, as low as 100 and 20 Nmm, in extraction torque that are associated with radiolucency, or as low as those that are manually extractable, are nearly the same between hydroxyapatite-coated and uncoated pins [11,14]. In contrast, FGF-CP-coated pins decrease the probability of impaired bone–pin interface strength without significant increases in average bone–pin interface strength. The average bone–pin interface strength for FGF-CP-coated pins (254 ± 132 Nmm (*n* = 20)) is not significantly higher than that of uncoated pins (227 ± 131 Nmm (*n* = 32) and 338 ± 269 Nmm (*n* = 40)). However, the probability of impaired bone–pin interface strength as low as 100 and 20 Nmm is lower in the FGF-CP pin group than in the un-coated pin group. It is suggested that Ti screws coated with FGF-CP layers are more reliable than uncoated pins for preventing impaired bone–pin interface strength that causes pin loosening.

## 5. Limitations

The current study is a retrospective study, and furthermore, a small number of patients were enrolled. In the future, prospective randomized controlled studies with increased enrollment of patients are needed. In addition, it may be necessary to show the relationship with bone mineral density in the future.

## 6. Conclusions

In the Weibull plot analysis of extraction torques, the slope of the regression line was higher in the FGF-CP group than in the uncoated pin group. Furthermore, the intercept of the regression line was significantly lower in the FGF-CP group than in the uncoated pin group. The two regression lines significantly differed. Therefore, Ti pins coated with FGF-CP layers have the potential to reduce the risk of impaired bone–pin interface strength in the external fixation of distal radius fractures.

## Data Availability

The datasets generated and analyzed during the current study are available from the corresponding authors upon reasonable request.

## Supplementary Materials

The following supporting information can be downloaded at: https://www.mdpi.com/article/10.3390/jcm13113040/s1, Figure S1: The Weibull plot of all the 40 extraction torques for uncoated pin groups.
