# Supplementary material for: Potential of Titanium Pins Coated with Fibroblast Growth Factor-2–Calcium Phosphate Composite Layers to Reduce the Risk of Impaired Bone–Pin Interface Strength in the External Fixation of Distal Radius Fractures
Source: J Clin Med. 2024 May 22;13(11):3040. doi: 10.3390/jcm13113040 (PMC11172767; doi:10.3390/jcm13113040)
Supplement: Supplementary file 1 [file jcm-13-03040-s001.zip › jcm-2954397-supplementary.pdf]

Journal of
Clinical Medicine

Supplementary Materials

Potential of Titanium Pins Coated with Fibroblast Growth Factor-2–Calcium Phosphate Composite Layers to Reduce the Risk of Impaired Bone–Pin Interface Strength in the External Fixation of Distal Radius Fractures

Hirotaka Mutsuzaki [1,2,*], Yohei Yanagisawa [3], Hiroshi Noguchi [4], Atsuo Ito [5] and Masashi Yamazaki [4]

[1] Center for Medical Science, Ibaraki Prefectural University of Health Sciences, Ami 300-0394, Japan
[2] Department of Orthopedic Surgery, Ibaraki Prefectural University of Health Sciences Hospital, Ami 300-0331, Japan
[3] Department of Emergency Medicine, University of Tsukuba, 1-1-1 Tennodai, Tsukuba 305-8575, Japan
[4] Department of Orthopaedic Surgery, University of Tsukuba, 1-1-1 Tennodai, Tsukuba 305-8575, Japan
[5] Health and Medical Research Institute, National Institute of Advanced Industrial Science and Technology (AIST), AIST Tsukuba Central 6, 1-1-1 Higashi, Tsukuba 305-8566, Japan
* Correspondence: mutsuzaki@ipu.ac.jp; Tel.: +81-29-888-4000

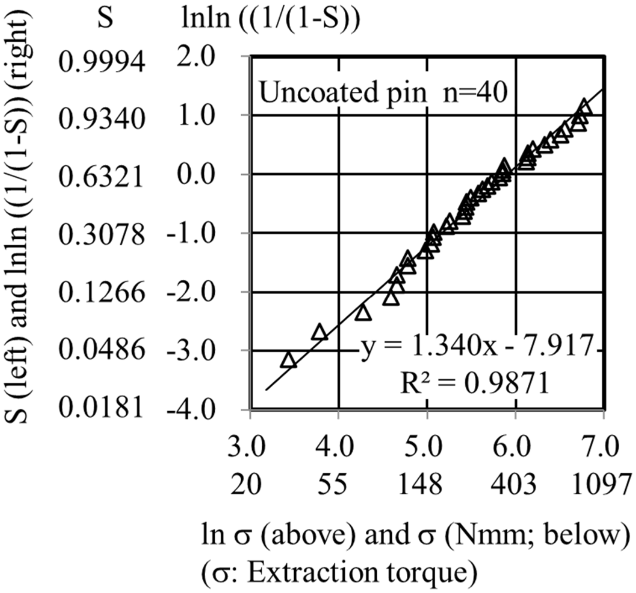

Figure S1. The Weibull plot of all the 40 extraction torques for uncoated pin groups.
